# The Bacillus of Tuberculosis

**Published:** 1882-10-20

**Authors:** 


**The Bacillus of Tuberculosis.**—The Allegemeine Medicinische Central Zeitung and the Berliner Klinische Wochenschrift for April 1st contains a brief reference to the discovery by Dr. Robert Koch of the bacillus of tuberculosis. This he has succeeded in cultivating through six or eight generations without diminishing its power. The bacillus, as far as has yet been proved, is identical both in men and animals. By inoculation, as well as by injection into the vessels, Koch has succeeded in producing acute miliary tuberculosis as well as cheesy processes in animals otherwise free from tuberculosis. This small bacillus grows very slowly, and is essentially different from all the other pathogenic bacteria and micrococci. The full text of the paper is promised for an early date.

Koch does not usually herald his discoveries until they are pretty well proved. Moreover, his late onslaught upon Grawitz for rushing in unseemly haste to unwarranted conclusions in regard to the inoculation of fungi furnishes an additional guaranty, if such be needed, that he is not likely to choose the present moment himself for abandoning those habits of exact method and care which have, as a rule, characterized his work hitherto. Our readers are likely to hear of this discovery again.—*Boston Med. and Surg. Jour.*

